# Genome-Wide Mapping of Growth-Related Quantitative Trait Loci in Orange-Spotted Grouper (*Epinephelus coioides*) Using Double Digest Restriction-Site Associated DNA Sequencing (ddRADseq)

**DOI:** 10.3390/ijms17040501

**Published:** 2016-04-06

**Authors:** Hui Yu, Xinxin You, Jia Li, Hankui Liu, Zining Meng, Ling Xiao, Haifa Zhang, Hao-Ran Lin, Yong Zhang, Qiong Shi

**Affiliations:** 1 BGI Education Center, University of Chinese Academy of Sciences, Shenzhen 518083, China; yuhui@genomics.cn (H.Y.); lijia1@genomics.cn (J.L.); 2 Shenzhen Key Lab of Marine Genomics, Guangdong Provincial Key Lab of Molecular Breeding in Marine Economic Animals, State Key Laboratory of Agricultural Genomics, BGI, Shenzhen 518083, China; youxinxin@genomics.cn (X.Y.); liuhankui@genomics.cn (H.L.); 3 State Key Laboratory of Biocontrol, Institute of Aquatic Economic Animals and Guangdong Provincial Key Laboratory for Aquatic Economic Animals, School of Life Sciences, Sun Yat-Sen University, Guangzhou 510275, China; mengzn@mail.sysu.edu.cn (Z.M.); xiaoling459@126.com (L.X.); 4 Marine Fisheries Development Center of Guangdong Province, Huizhou 510610, China; zhhaifa812@hotmail.com; 5 South China Sea Bio-Resource Exploitation and Utilization Collaborative Innovation Center, Guangzhou 510275, China; 6 College of Life Sciences, Shenzhen University, Shenzhen 518060, China

**Keywords:** quantitative trait loci (QTL), genetic linkage map, growth-related genes, ddRADseq, orange-spotted grouper (*Epinephelus coioides*)

## Abstract

Mapping of quantitative trait loci (QTL) is essential for the discovery of genetic structures that related to complex quantitative traits. In this study, we identified 264,072 raw SNPs (single-nucleotide polymorphisms) by double digest restriction site associated DNA sequencing (ddRADseq), and utilized 3029 of these SNPs to construct a genetic linkage map in orange-spotted grouper (*Epinephelus coioides*) using a regression mapping algorithm. The genetic map contained 24 linkage groups (LGs) spanning a total genetic distance of 1231.98 cM. Twenty-seven significant growth-related QTLs were identified. Furthermore, we identified 17 genes (*fez2*, *alg3*, *ece2*, *arvcf*, *sla27a4*, *sgk223*, *camk2*, *prrc2b*, *mchr1*, *sardh*, *pappa*, *syk*, *tert*, *wdrcp91*, *ftz-f1*, *mate1* and *notch1*) including three (*tert*, *ftz-f1* and *notch1*) that have been reported to be involved in fish growth. To summarize, we mapped growth-related QTLs in the orange-spotted grouper. These QTLs will be useful in marker-assisted selection (MAS) efforts to improve growth-related traits in this economically important fish.

## 1. Introduction

Orange-spotted grouper, *Epinephelus coioides* (Epinephelinae, Serranidae), a protogynous hermaphrodite species, is mainly distributed in the Indo-West-Pacific region [1]. It is an economically important aquaculture species in many Asian countries, especially in China, due to its desirable taste and as a source of nutrition [2]. Orange-spotted grouper has become an important edible fish species in live fish markets and is an important cultured fish for sale in markets in southeast China. Market demand for the orange-spotted grouper has prompted the development of fish families and populations characterized by lower food consumption and higher growth rates. The application of marker-assisted selection (MAS) to orange-spotted grouper will be useful to improve important traits, such as feed conversion rate, meat quality and disease resistance. These traits have a major effect on productivity and profitability.

High-density genetic linkage maps and quantitative trait loci (QTL) mapping provide a framework for the MAS program. At present, genetic linkage maps have been constructed in over 28 fish species and economic traits have been mapped in at least 13 fish species [3]. These include the Nile tilapia (*orechromis niloticus*) [4], Atlantic salmon (*Salmo salar*) [5], rainbow trout (*Oncorhynchus mykiss*) [6], and channel catfish (*Ictalurus punctatus*) [7]. Microsatellite-based linkage maps have been reported for the white grouper (*Epinephelus aeneus*) [8] and kelp grouper (*Epinephelus bruneus*) [9]. The density of the genetic linkage maps is typically determined by the marker type. Single-nucleotide polymorphisms (SNPs), the most stable and abundant form of genetic marker, are the ideal marker type for construction of high-density genetic linkage maps [10,11,12]. The advent of next-generation sequencing (NGS) has made it possible to discover thousands of SNPs dispersed throughout the genome in a single procedure, even if little genetic information is available on the species [13]. A SNP-based high-density genetic linkage map has been constructed for orange-spotted grouper by our lab [14], but QTL mapping was not reported.

This is the first report of a SNP-based high-resolution genetic linkage map of the orange-spotted grouper developed using double digest restriction site associated DNA sequencing (ddRADseq). We also report the identification of 27 significant growth-related QTLs and 17 corresponding genes in this economically important fish.

## 2. Results

### 2.1. Sequencing and Genotyping

In total, 8.2 × 10^8^ raw 90 bp reads were generated and 7.3 × 10^8^ clean reads were retained after removal of low-quality raw reads. These high-quality reads were partitioned into ddRAD tags. The average number of ddRAD tags in each individual was 10,428,571. For the analysis of the F_1_ mapping population, a total of 264,072 candidate ddRAD loci were inferred from 68 individuals. If both parents were homozygous, the SNPs were eliminated. The SNPs that were significant segregation distortion based on Mendelism by Chi-square analysis were also eliminated. Ultimately, 26,661 SNPs were obtained (see detailed information in Table S1).

### 2.2. Genetic Linkage Map

After construction of the genetic linkage map using the 26,661 SNPs, we obtained 24 linkage groups (LGs), which is consistent with the haploid chromosome number of the orange-spotted grouper [15]. A total of 3029 SNPs were successfully mapped to the 24 LGs (Table S2 and Figure S1) after discarding contradictory SNPs. These LGs spanned a total genetic distance of 1231.98 cM with the length of each LG ranging from 25.43 cM (LG18) to 111.47 cM (LG8). The average genetic length of the LGs was 51.20 cM. The number of SNPs in the different LGs ranged from 94 (LG12) to 150 (LG22) with an average number of 126. Detailed information about loci and SNPs is summarized in Table S3.

### 2.3. Growth Trait–Associated Quantitative Trait Loci (QTL) and Related Genes

**Figure 1:**
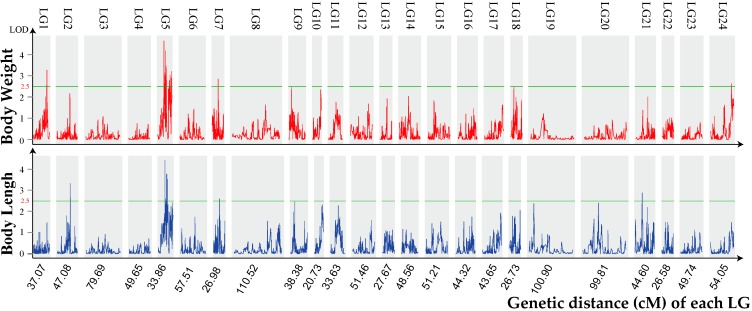
The genetic location of growth-related (body weight and body length) quantitative trait loci (QTLs) on the 24 linkage groups (LGs) of orange-spotted grouper. The green orizontal lines indicate the cutting threshold of logarithm of odds (LOD) at 2.5. The red and blue coloration represents the traits of body weight and body length, respectively.

In total, 27 significant QTLs for growth traits were found to be distributed on LG1, LG5, LG7, LG21 and LG24 of the orange-spotted grouper (Table 1 and Figure 1). These included 18 QTLs for body weight (BW) and nine QTLs for body length (BL). The majority of these QTLs clustered together on their respective LGs. Especially, QTLs (qLG5-1, qLG5-2, qLG5-3, qLG5-4, qLG5-5, qLG5-6, qLG5-7, qLG5-8, qLG5-9, qLG5-10, qLG5-11, qLG5-12, qLG5-13, qLG5-14, qLG5-15, qLG5-16, qLG5-17, qLG5-18, qLG5-19, qLG5-20 and qLG5-21) were found to be clustered in a narrow region (15.1–33.3 cM) on the LG5.

Among these, the logarithm of odds (LOD) value of qLG5-1 for body weight, which accounts for 24.3% of the phenotypic variation, located at 15.1–15.6 cM is the highest (4.7), while qLG5-16 located at 15.1–15.4 cM for the trait of body length had the LOD value of 4.4 and it explains 23.3% of the phenotypic variation. The other QTLs on LG5 for BW were detected at positions 17.0–17.5, 17.8–18.0, 18.7–19.4, 19.9–20.2, 20.4–20.8, 27.4–27.9, 28.1–28.3, 29.4–29.7, 32.2–32.4 and 33.0–33.3 cM, with LOD values of 2.6–4.2, accounting for 14.6%–29.1% of the phenotypic variation. The other BL QTLs on LG5 were detected at positions 17.0–17.1, 19.2–19.3, 19.9–20.2, 20.4–20.8 cM, with LOD values of 2.6–3.8. These accounted for 14.5%–22.2% of the phenotypic variation. The QTLs for BW and BL were distributed across all 24 LGs. However, there was only one QTL for BW on LG1 and one QTL for BL on LG2.

Seventeen genes, including fasciculation and elongation protein ζ-2-like (*fez2*), Dol-P-Man: Man(5)GlcNAc(2)-PP-Dol α-1,3-mannosyltransferase (*alg3*), endothelin converting enzyme 2 (*ece2*), armadillo repeat gene deleted in velocardiofacial syndrome (*arvcf*), solute carrier family 27 (fatty acid transporter), member 4 (*sla27a4*), tyrosine-protein kinase SgK223 (*sgk223*), calcium/calmodulin-dependent protein kinase 2 (*camk2*), proline-rich coiled-coil 2B (*prrc2b*), melanin-concentrating hormone receptor 1 (*mchr1*), sarcosine dehydrogenase (*sardh*), pregnancy-associated plasma protein-A (*papp-a*), spleen tyrosine kinase (*syk*), telomerase reverse transcriptase (*tert*), WD repeat-containing protein 91-like (*wdrcp91*), ftz transcription factor 1 (*ftz-f1*), multidrug and toxin extrusion protein 1-like (*mate1*) and neurogenic locus notch homolog protein 1-like (*notch1*), were identified (Table 2). Among these, 12 genes (*arvcf*, *sla27a4*, *sgk223*, *camk2*, *prrc2b*, *mchr1*, *sardh*, *papp-a*, *syk*, *tert*, *wdrcp9* and *ftz-f1*), corresponding to nine QTLs, were clustered on LG5. Two genes (*alg3* and *ece2*), representing a single QTL, were distributed on LG2. Three genes including *fez2*, *mate4* and *notch1* mapped as a single QTL to LG1, LG7 and LG21, respectively.

## 3. Discussion

In this study, we applied the technology of ddRADseq, an extension of RADseq [16]. This technique has the advantage of providing improved efficiency and robustness by utilizing two restriction enzymes (a frequently-cutting enzyme and a rare-cutting enzyme) [16,17,18,19]. Under-sampling in read count was a constant question resulting from biased read representation in pooled sequencing experiments among individual samples. ddRADseq increases its sturdiness compared to RADseq [20,21,22]. ddRAD-based genetic maps of Nile tilapia (*Oreochromis niloticus*) [23], Japanese eel (*Anguilla japonica*) [17], Midas cichlids (*Amphilophus spp.*) [24,25], and Eurasian perch (*Perca fluviatilis L.*) [19] have been successfully constructed. Using ddRADseq, 264,072 candidate RAD loci were inferred, and 3029 high-quality SNPs were retained after a series of filtering. The number of mapped genetic markers in the present study was less than that in our previous report [14]. However, ddRAD-seq was better than MSG (multiplexed shotgun genotyping) in the detection of SNPs because MSG with a stringent methodology may overlook some significant loci [26]. There were fewer markers mainly due to the use of a small F_1_ full-sib population.

QTL mapping is considered an efficient strategy for analyzing complex quantitative traits in a variety of fish species. For example, 17 QTLs were detected for the traits of body size (body length and weight) of Chinook salmon (*Oncorhynchus tshawytscha*) [27] and 11 QTLs were found to be related to the body shape of lake cichlid fishes [25]. Additionally, growth-related QTLs had been identified in Atlantic salmon (*Salmo salar*) [5] and Arctic charr (*Saivelinus alpinus*) [28,29]. Recently, a genetic linkage map of kelp grouper had been constructed using simple sequence repeat (SSR) markers, and growth-related (body weight and total length) QTL analysis was performed [30]. The high-resolution genetic map consisted of 714 SSR markers. One major growth-related QTL and several putative QTLs were detected.

The previous data were from the family without phenotype information and were used to construct a SNP-based high-density genetic linkage map. However, our current mapping data from ddRADseq were applied to construct a genetic map and perform QTL analysis based on the available phenotypic information. Twenty-seven QTLs associated with growth (body weight or body length) were identified and found to be distributed on six LGs (LG1, LG2, LG5, LG7, LG21 and LG24). Interestingly, 21 out of the 27 QTLs were concentrated on LG5 within a narrow region (15.1–33.3 cM). These had the highest LOD value (4.7), accounting for 14.5%–29.1% of the phenotypic variation. The small physical and genetic distance among QTLs within the cluster suggested that the cluster would be highly effective for future marker-assisted selection.

Target genes associated with the growth traits of orange-spotted grouper have been previously studied [31]. We compared our QTLs with the scaffold assembly and annotation of the orange-spotted grouper reference genome [32], and discovered a total of 17 genes within the QTL regions. Three of these genes (*notch1*, *ftz-f1* and *tert*) have been reported to be involved in fish growth [33,34,35]. The *notch1* signaling gene plays a role in the notochord development of zebrafish [32]. The development of the notochord is strongly linked with body length. Growth was correlated with gonadal development and sex change in groupers [36]. In tilapia, *ftz-f1* is involved in the development of adrenal-gonadal and sex determination and its transcripts were only expressed in the gonads and kidneys [34]. Phylogenetic analysis of the tilapia *ftz-f1* indicated that it was highly conserved among other teleosts. In addition, the orange-spotted grouper is a protogynous hermaphroditic and the expression of *ftz-f1* in gonads is influenced by the development of the gonad [37]. The gene of *tert* is associated with the physiological aging of teleosts and plays a major part in the cell process of proliferation, differentiation and tumorigenesis [35]. Expression of *tert* was also significantly correlated with muscle telomerase activity (TA) in the skeletal muscle of many fish species [38]. TA has a major effect on the growth rate of orange-spotted grouper. Fourteen additional genes (Table S4) were also mapped to growth-related QTLs, but their exact roles with regard to fish growth remain to be determined.

## 4. Materials and Methods

### 4.1. Sample Preparation

Parent fishes (orange-spotted grouper) were captured from the South China Sea in Hainan. One female and one male, with desirable properties such as the coefficient of mature (stage IV) gonad, genetically diverse and highly heterozygous, high vigor, *etc.*, were selected. An F_1_ full-sib family was generated by crossing the female and male fish at the Daya Bay Seawater Fish Farm in Huizhou, Guangdong Province, China. The F_1_ progeny were raised under a natural photoperiod, at the water temperature 28 °C. They were fed according to management practices of the fishery. Fin clips of the parents and 68 offspring (four months of age) were collected and stored in absolute ethanol at −20 °C before use. Phenotypic information on the F_1_ progeny is summarized in Table S5. Phenotypic correlations were tested with the Pearson correlation coefficient, and the results indicated a significant correlation (*p* < 0.01) between body weight and body length (Table S6). Genomic DNA was extracted using a standard phenol-chloroform protocol [39]. The quality of all DNA samples was evaluated by Qubit Fluorometer (Invitron, Waltham, MA, USA). Electrophoresis was conducted on 0.6% agarose gels. All the experiments were carried out in accordance with the guidelines of the Animal Ethics Committee and were approved by the Institutional Review Board on Bioethics and Biosafety of BGI (No. FT14015, 12 March 2014).

### 4.2. ddRAD Library Construction and Sequencing

The ddRADseq library was prepared using a previously reported protocol [16] with some modifications. Briefly, the workflow included double-digestion, ligation reaction, pooling, purification, and amplification. The double-digestion and ligation reactions were prepared as 30- and 40-µL reactions (see more details in Tables S7 and S8), respectively. One µL of the ligation products from each sample was pooled 24 samples in a new centrifuge tube, respectively. The DNA fragments were purified (300–700 bp) were isolated on a 3% agarose gel, and then extracted with the QIA quick Gel Extraction Kit (Qiagen, Hilden, Germany). All the samples were amplified by 12 cycles of PCR in the 50-µL reaction volumes containing 2-µL purified of the PCR products, 20-µL Master Mix, 2-µL primers (1-µL each) and 26-µL ddH_2_O. The amplified products were purified using the QIA quick PCR Purification Kit (Qiagen) and checked the quality (concentration and purity) of DNA products via an Agilent 2100 Bioanalyzer (Agilent, Santa Clara, CA, USA). Finally, library sequencing was performed using a Hiseq 2000 platform (Illumina, San Diego, CA, USA) with 90-bp pair-end reads.

### 4.3. SNP (Single-Nucleotide Polymorphism) Calling and Genotyping

We discarded the Illumina short reads without sample-specific barcodes and restriction enzyme motifs. Subsequently, we used Soapsnp [40] to sort retained reads into loci and to genotype them. Lastly, we aligned the clean reads of the two parents to the previously assembled orange-spotted grouper genome [32] to eliminate monomorphic DNA sequences and then added the reads containing SNPs. SNPs were selected as described previously [41]. Regions with these putative SNPs were defined as reference SNP regions, and then the clean reads of progeny samples were aligned to the reference SNP regions. As noted earlier, the parental genotypes determined the genotypes of the individual offspring [42].

### 4.4. High-Density Genetic Map Construction

The SNPs that at least one parent was heterozygous and had high quality of genotype calls were retained for genetic linkage analysis. Those SNPs with significant segregation distortion (*p* < 0.01, *x*^2^ test) were discarded. We used JoinMap 4.1 (Kyazma B.V., Wageningen, Gelderland, Netherlands) to conduct the genetic map construction with the genotypic data from the F_1_ mapping population. The logarithm of odds (LOD) threshold was set as from two to 15. It was adopted as indicator to cluster analysis using regression mapping algorithm. Map distances were calculated using the Kosambi mapping function in centiMorgans (cM). All the SNPs were arranged and grouped into 24 linkage groups (LGs). Graphics of the linkage groups were generated using custom perl script.

### 4.5. QTL Mapping

We used WinQTLCart2.5 software (Wang S., Basten C.J. and Zeng Z.-B., Raleigh N.C., USA) to perform the QTL analyses with the method of composite interval mapping (CIM) [43]. The CIM method was executed using Model 6 with parameters of five control markers, a 10-cM window size and backward regression. We used 1000 permutations and the *p*-value (0.05) of whole genome-wide significance to determine the threshold level of likelihood ratio (LR) (11.5) or LOD (2.5). The LR peak location and surrounding district of every QTL was determined. Phenotypic variation resulting from growth-related QTLs was also calculated by the software.

### 4.6. Identification of the Genes on Growth Traits Associated QTLs

The genes on growth-associated QTLs were identified via following steps. First we identified the orange-spotted grouper genome scaffolds [32] from these QTL regions. Subsequently, we mapped the corresponding gene identities (IDs) based on their position on the scaffolds. Finally, we retrieved the homologous genes from the orange-spotted grouper gene annotation file [32].

## 5. Conclusions

A high-density genetic linkage map with a total genetic distance of 1231.98 cM was constructed for the orange-spotted grouper using the ddRADseq method. Twenty-seven growth-related QTLs were identified by QTL mapping and corresponding 17 genes were discovered. These results will provide a valuable resource in future marker-assisted molecular breeding studies of this economically important fish species.

## Figures and Tables

**Table 1:** The information of growth-related quantitative trait loci (QTLs) for body weight (BW) and body length (BL) in orange-spotted grouper.

QTL	Trait	Genetic Position(cM)	Logarithm of Odds (LOD)	*R*^2^ (%)
qLG1	BW	30.8–31.0	3.3	19.6
qLG2	BL	29.8–30.2	3.3	21
qLG5_1	BW	15.1–15.6	4.7	24.3
qLG5_2	BW	17.0–17.5	3.1	17.1
qLG5_3	BW	17.0–17.5	2.8	16.5
qLG5_4	BW	17.8–18.0	2.9	15.8
qLG5_5	BW	18.7–19.4	3.2	19.1
qLG5_6	BW	18.7–19.4	4.2	24.6
qLG5_7	BW	19.9–20.2	3.5	20.8
qLG5_8	BW	20.4–20.8	3.4	20
qLG5_9	BW	27.4–27.9	2.6	21.5
qLG5_10	BW	27.4–27.9	2.8	24.5
qLG5_11	BW	28.1–28.3	2.8	28.3
qLG5_12	BW	29.4–29.7	2.7	14.6
qLG5_13	BW	29.4–29.7	3	29.1
qLG5_14	BW	32.2–32.4	3.2	17.6
qLG5_15	BW	33.0–33.3	2.9	15.6
qLG5_16	BL	15.1–15.4	4.4	23.3
qLG5_17	BL	17.0–17.1	2.6	14.5
qLG5_18	BL	19.2–19.3	3.8	20.5
qLG5_19	BL	19.9–20.2	3.4	20.3
qLG5_20	BL	19.9–20.2	3.8	22.2
qLG5_21	BL	20.4–20.8	3.5	20.8
qLG7_1	BW	14.4–14.5	2.8	16.8
qLG7_2	BL	14.4–14.5	2.6	15.1
qLG21	BL	16.0–16.5	2.9	15.5
qLG24	BW	48.8–49.0	2.6	14.4

**Table 2:** Genes in the growth-related QTL of orange-spotted grouper.

QTL	Gene ID	Gene Description	Gene Symbol	Physical Position
qLG1	Eco_gene_10012941	fasciculation and elongation protein ζ-2-like	*fez2*	scaffold416
qLG2	Eco_gene_10013595	Dol-P-Man: Man(5)GlcNAc(2)-PP-Dolα-1,3-mannosyltransferase	*alg3*	scaffold438
	Eco_gene_10013597	endothelin converting enzyme 2	*ece2*	scaffold438
qLG5_1	Eco_gene_10014238	armadillo repeat gene deleted in velocardiofacial syndrome	*arvcf*	scaffold468
qLG5_3	Eco_gene_10004531	solute carrier family 27 (fatty acid transporter), member 4	*slc27a4*	scaffold1517
	Eco_gene_10005552	tyrosine-protein kinase SgK223	*sgk223*	scaffold1706
qLG5_4	Eco_gene_10002069	calcium/calmodulin-dependent protein kinase 2	*camk2*	scaffold1180
qLG5_5	Eco_gene_10005555	proline-rich coiled-coil 2B	*prrc2b*	scaffold1706
	Eco_gene_10000893	melanin-concentrating hormone receptor 1	*mchr1*	scaffold1067
qLG5_19	Eco_gene_10005043	sarcosine dehydrogenase	*sardh*	scaffold1600
qLG5_8	Eco_gene_10018102	pregnancy-associated plasma protein A	*pappa*	scaffold675
qLG5_9	Eco_gene_10014809	spleen tyrosine kinase	*syk*	scaffold491
qLG5_11	Eco_gene_10002109	telomerase reverse transcriptase	*tert*	scaffold1187
qLG5_12	Eco_gene_10005999	WD repeat-containing protein 91-like	*wdrcp91*	scaffold1804
	Eco_gene_10000470	ftz transcription factor 1	*ftz-f1*	scaffold1023
qLG7	Eco_gene_10021611	multidrug and toxin extrusion protein 1-like	*mate1*	scaffold919
qLG21	Eco_gene_10003094	neurogenic locus notch homolog protein 1-like	*notch1*	scaffold130

## Supplementary Materials

Supplementary materials can be found at http://www.mdpi.com/1422-0067/17/4/501/s1.
