# Supplementary material for: Genome-Wide Mapping of Growth-Related Quantitative Trait Loci in Orange-Spotted Grouper (Epinephelus coioides) Using Double Digest Restriction-Site Associated DNA Sequencing (ddRADseq)
Source: Int J Mol Sci. 2016 Apr 6;17(4):501. doi: 10.3390/ijms17040501 (PMC4848957; doi:10.3390/ijms17040501)
Supplement: Supplementary file 1 [file ijms-17-00501-s001.zip › ijms-123090-Supplementary Materials/ijms-123090-Supplementary.pdf]

Supplementary Materials: Genome-Wide Mapping of Growth-Related Quantitative Trait Loci in Orange-Spotted Grouper (*Epinephelus coioides*) Using Double Digest Restriction Site Associated DNA Sequencing (ddRADseq)

Hui Yu, Xinxin You, Jia Li, Hankui Liu, Zining Meng, Ling Xiao, Haifa Zhang, Hao-Ran Lin, Yong Zhang and Qiong Shi

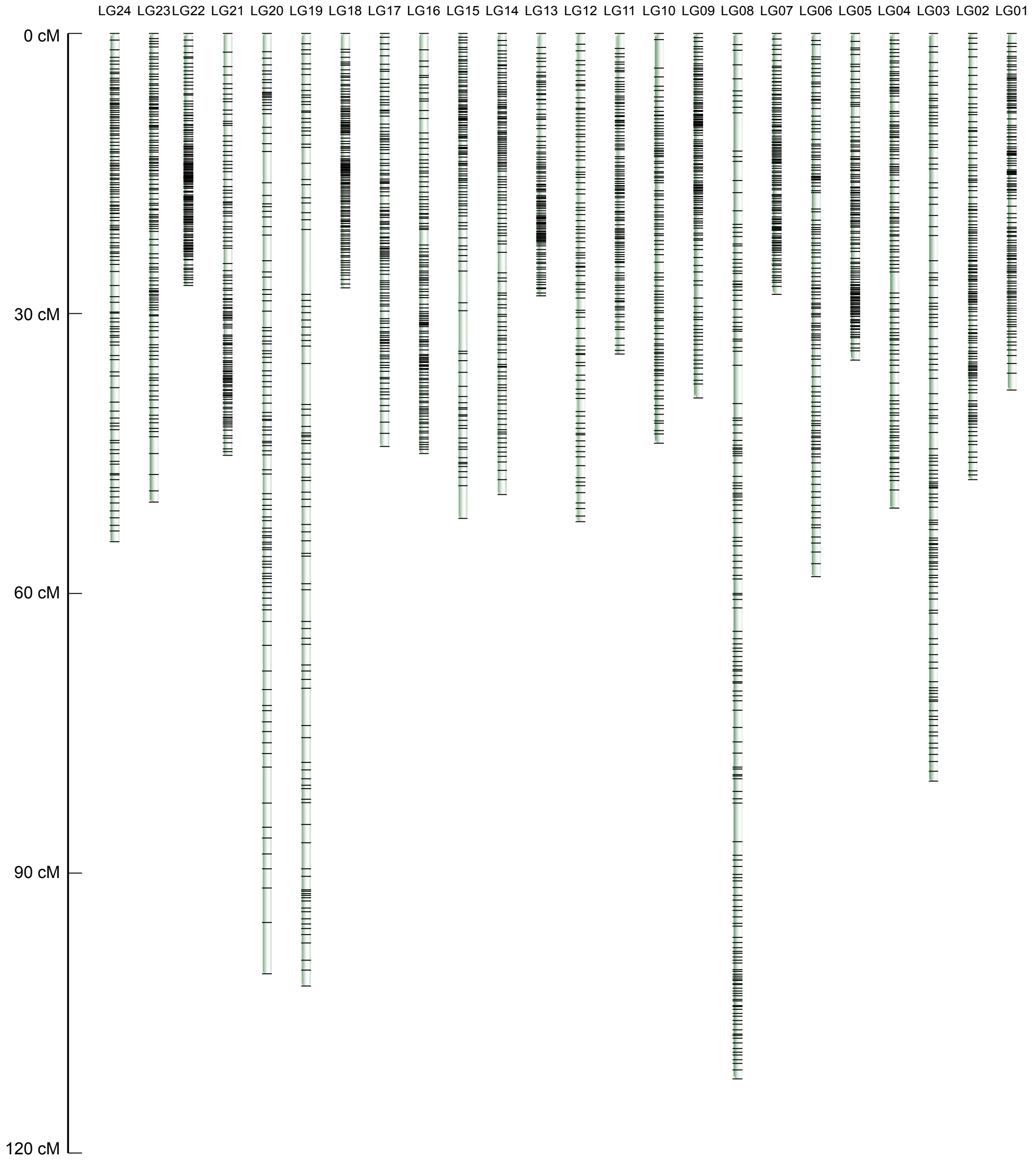

Figure S1. The distribution of SNPs (single-nucleotide polymorphisms) on the 24 linkage groups (LGs).

Table S2. The genetic linkage map of orange-spotted grouper.

Chromosome	Marker Number	Distance (cM)
LG1	131	38.003
LG2	142	47.544
LG3	128	79.725
LG4	117	50.623
LG5	130	34.838
LG6	127	57.911
LG7	121	27.802
LG8	169	111.465
LG9	133	38.86
LG10	103	43.686
LG11	116	34.196
LG12	94	52.042
LG13	115	26.459
LG14	131	48.457
LG15	123	51.329
LG16	132	43.051
LG17	127	44.031
LG18	133	25.432
LG19	103	101.569
LG20	115	100.273
LG21	129	44.994
LG22	150	25.537
LG23	130	49.952
LG24	130	54.199
Average	126	51.199
Total	3029	1231.978

Table S4. The putative roles of 14 genes.

Gene Symbol	Gene Description	Gene ID	Putative Role	Reference
wdrcp91	WD repeat-containing protein 91-like	Eco_gene_10005999	unreported	–
prrc2b	proline-rich coiled-coil 2B	Eco_gene_10005555	unknown	[44]
FEZ2	fasciculation and elongation protein zeta-2-like	Eco_gene_10012941	related genes to age in humans	[45]
alg3	Dol-P-Man:Man(5)GlcNAc(2)-PP-Dolα-1,3-mannosyltransferase	Eco_gene_10013595	related genes to growth, protein secretion, pigment production and sporulation in *Aspergillus niger*	[46]
ECE2	endothelin converting enzyme 2	Eco_gene_10013597	related genes to the generation of novel neuropeptides, Alzheimer's disease, the development of murine cardiac	[47–49]
ARVCF	armadillo repeat gene deleted in velocardiofacial syndrome	Eco_gene_10014238	related genes to neurodevelopment	[50]
SLC27A4	solute carrier family 27 (fatty acid transporter), member 4	Eco_gene_10004531	related to the lean percentage and loin eye area of pig	[51]
SGK223	tyrosine-protein kinase SgK223	Eco_gene_10005552	related to the signaling pathway of pancreatic ductal adenocarcinoma (PDAC) progression in humans	[52]
CAMK2	calcium/calmodulin-dependent protein kinase 2	Eco_gene_10002069	related to mitochondrial adenosine triphosphate (ATP) synthesis activity	[53]
MCHR1	melanin-concentrating hormone receptor 1	Eco_gene_10000893	related to the control of feeding, stress and mood	[54,55]
SARDH	sarcosine dehydrogenase	Eco_gene_10005043	related to one-carbon metabolism and cellular invasion	[56]
PAPP-A	pregnancy-associated plasma protein-A	Eco_gene_10018102	related to dissolving fat, gestational diabetes mellitus, obstetric cholestasis	[57–59]
SYK	spleen tyrosine kinase	Eco_gene_10014809	related to proximal immunoreceptor signaling, keratinocyte terminal differentiation, the survival and migration of multiple myeloma cells	[60,61]
MATE1	multidrug and toxin extrusion protein 1-like	Eco_gene_10021611	related to flutamide pharmacokinetics of the peripheral blood cell in the process of the liver injury development	[62]

Table S6. Pearson correlation coefficients for body weight and body length.

Growth-Related Traits	No. of Progeny	Body Weight (g)	Body Length (cm)
Body Weight (g)	68	1	0.969 **
Body Length (cm)	68	0.969 **	1

**: Significant correlation at 0.01 level (two-tailed).

Table S7. The components of 30 μL double-digestion reaction system.

Reagent	Volume (μL)
DNA template	20
Taq I *	1
10X Taq I buffer	3
Msp I **	1
10X Msp I buffer	3
Nuclease-free water	1

*: 15 U/μL, restriction enzyme cleavage site 5′ T^CGA 3′ (Thermo Scientific, Waltham, MA, USA);
**: 15 U/μL, restriction enzyme cleavage site 5′ C^CGG 3′ (Thermo Scientific, Waltham, MA, USA).

Table S8. The components of 40 μL ligation reaction system.

Reagent	Volume (μL)
enzyme-digested products	30
unique adapter	1
10X Taq I buffer	1
10X Msp I buffer	1
T4 DNA ligase	2
dNTP	0.4
ddH_2O	4.6
